# Morphological analysis of dendrites and spines by hybridization of ridge detection with twin support vector machine

**DOI:** 10.7717/peerj.2207

**Published:** 2016-07-20

**Authors:** Shuihua Wang, Mengmeng Chen, Yang Li, Ying Shao, Yudong Zhang, Sidan Du, Jane Wu

**Affiliations:** 1 School of Electronic Science and Engineering, Nanjing University, Jiangsu, China; 2 School of Computer Science and Technology, Nanjing Normal University, Nanjing, Jiangsu, China; 3 Department of Neurology, Northwestern University School of Medicine, Chicago, USA; 4 State Key Laboratory of Brain and Cognitive Science, Institute of Biophysics, Chinese Academy of Sciences, Beijing, China; 5 School of Psychology, Nanjing Normal University, Nanjing, Jiangsu, China

**Keywords:** Neuron, Dendritic spine, Ridgelet detection, Twin Support vector machine

## Abstract

Dendritic spines are described as neuronal protrusions. The morphology of dendritic spines and dendrites has a strong relationship to its function, as well as playing an important role in understanding brain function. Quantitative analysis of dendrites and dendritic spines is essential to an understanding of the formation and function of the nervous system. However, highly efficient tools for the quantitative analysis of dendrites and dendritic spines are currently undeveloped. In this paper we propose a novel three-step cascaded algorithm–RTSVM— which is composed of ridge detection as the curvature structure identifier for backbone extraction, boundary location based on differences in density, the Hu moment as features and Twin Support Vector Machine (TSVM) classifiers for spine classification. Our data demonstrates that this newly developed algorithm has performed better than other available techniques used to detect accuracy and false alarm rates. This algorithm will be used effectively in neuroscience research.

## Introduction

The dendrite is defined as the branched projection of a neuron. The dendritic spine is described as neuronal protrusions attached to the neuronal dendrites (Wang et al., 2015). The structure of the spine is composed of a small head which is connected to the shaft of the dendrite by its thin neck. They work by assisting the transmission of electrical signals to the neuronal soma and providing essential energy storage for the synapses. Statistics show that the length of the spine is usually between 0.5–2 µm with some measurements of the Cornu Ammonis three (CA3) region of the hippocampus measuring up to 6 µ. Volume ranges from 0.01 µm^3^ to 0.8 µ^3^ (Zito & Murthy, 2002).

**Figure 1:**
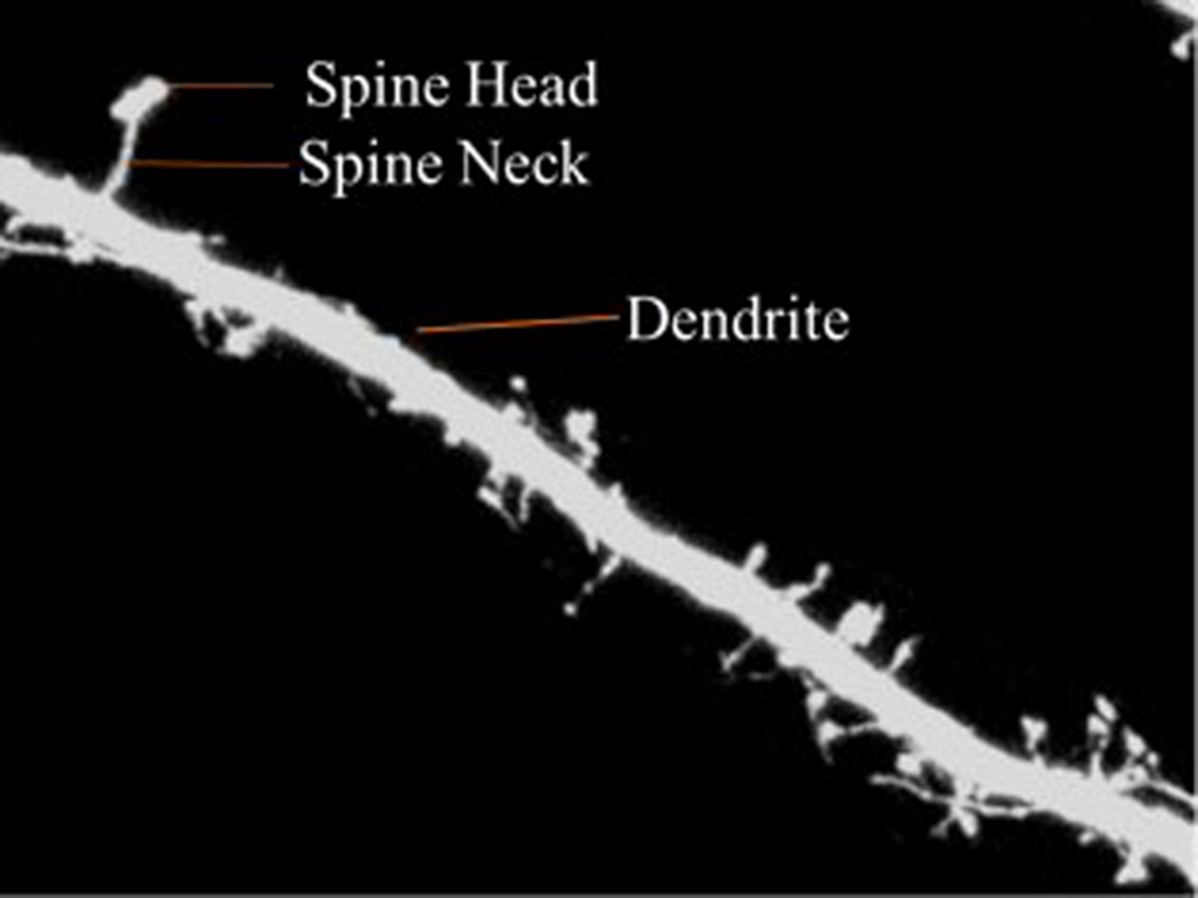
Dendritic spine.

Dendritic spines are categorized into three types according to shape—thin, stubby or mushroom shaped. The morphology of the dendritic spines varies over time (Engert & Bonhoeffer, 1999; Yuste & Bonhoeffer, 2001). Research shows that the morphology of dendritic spines had a strong relationship to neuron function (Johnston & Wu, 1994; Krichmar et al., 2002; Mainen & Sejnowski, 1996). For example, researchers have traditionally used the relationship between the morphological and functional classes of cat retinal ganglion cells to illustrate the interaction between neuronal shape and function (Sun, Li & He, 2002). Characterization of dendritic spinal structures has the potential to profoundly impact biological research and an understanding of neuronal morphology has important, beneficial implications and can be instructive in the treatment of neurological and psychiatric disorders such as Attention-deficit hyperactivity disorder (ADHD), autism, cognitive disorders and Alzheimer’s and Parkinson’s diseases. Figure 1 is provided to illustrate the dendritic spine and dendrite.

Within the last several decades, numerous automatic and semi-automatic neuron analysis methods have been proposed, such as ImageJ, Neuron Studio etc. For example, SynD, proposed by Schmitz et al. (2011) was a semi-automatic image analysis software designed specifically for immuno-fluorescent imagery. It was based on the principal that neuron structure information is obtained in different channels. Jie et al. (2007) proposed a pipeline method which included an adaptive threshold that could improve segmentation performance over that of the global threshold method. Following this an efficient backbone extraction method was introduced. Detached spines were detected based on the signal noise ratio (SNR) values as well as local dendrite morphology estimates. Zhang et al. (2007) proposed use of curvilinear structure detection in the identification of dendritic spines and backbones. The result is exclusion of pseudo-spines among the attached spines based on linear discriminate analysis. Rodriguez et al. (2008) introduced a new system for spine detection, shape analysis and classification. The spinal analysis module in Alfredo et al.’s paper posited that the model of the dendritic tree could be identified as a series of nodes of specified diameter. The spine detection was therefore based on voxel clustering. Fan et al. (2009) presented their method in the vivo mouse models. They isolated the medical axes of dendritic backbones and attached spines based on the curvilinear structure detector. They then used the adaptive local binary fitting energy level set model to determine the dendritic boundary and the central line of the curvilinear detector in the initial samples. Spinal growth was tracked based on the graph homomorphism of dual image graph structures at different times. In order to discriminate between the androgynous and exdrogynous effects on spineogensis. Hideo et al. (2011) proposed an automatic analysis method of spines based on confocal laser microscopic imaging. They developed a new method based on the geometrical features, which used scale free shape dependent analysis combined with manual correction. He, Xue & Wong (2012) proposed a nonlinear degeneration equation (NDE) method. The morphological differences between the dendrites and spines was enhanced by the NDE method due to differences in pixel shrinkage rates between spines and dendrites. For the classification of different types of spines, Gaussian curvatures and the biomimetic pattern recognition theory were used. Su et al. (2014) proposed to use the directional morphological filter (DMF) and shortest path (SP). Extractions were refined by DMF until the desired results were achieved. Next, SP was used to locate the dendritic boundary in order to determine the beginning points of the spines. Finally, the spines were segmented from the dendrites outside of the extracted boundary. Marker-controls were then used to split the attached spines.

The limitations of these algorithms, however, are that they perform well on specific images but poorly on new query images from different types of microscopies. In addition, existing manual methods were time consuming, costly, and irreproducible. Increased dimensionality (Zhang et al., 2016), the result of the immense size of the imaging data, necessitated the design of more efficient automated spinal analysis.

Therefore, we propose a novel, robust automatic three step cascaded method, composed of ridge backbone detection, Hu moment invariants (HuMI) for feature extraction and twin SVM for spinal type classification. This paper is thus organized into the following sections: Section 1: Research importance and impact statement and an introduction of current research and progress. Section 2: Methodology used in the paper, including image preprocessing, ridge-based backbone extraction, Hu moments based feature extraction and Twin SVM based classification. Section 3: Results and discussion of our proposed algorithm. Section 4: Conclusion and discussion of ongoing work.

## Method

Our proposed approach begins with a morphological operation to remove the small jiggers as the noise for the backbone extraction. Next, the curvilinear structure detector is implemented to isolate the dendritic backbone. The dendritic boundary is then obtained by creating sharp density differentiation. Finally, Twin SVM classifies the spines as pseudo or positive—with positive spines being sub-classified by shape (mushroom, stubby or thin (Nimchinsky, Sabatini & Svoboda, 2002)) according to their Hu Moments invariants features (Hu & Mingkuei, 1962). Figure 1 shows a flowchart of our proposed approach.

### Image acquisition

The cortical neurons used for imaging initiated from the embryonic (E) 18th day rat. Neurons were cultured *in vitro* until day 22. Finally, Green fluorescent protein (GFP) was transfected using Lipofectamine2000 by Lipofectamine 2000 and neuron was imaged on day 24 by Leica SP5 confocal laser scanning microscopy (CLSM) with a 63X magnification. The size of the image is 1,024 × 1,024 with a resolution of 0.24 µm/pixel at the confocal layer. We added a white line in the figures to indicate the scale bar of 10 micron by 41 pixels.

As the images were captured as a Z-stack series a 3D image stack was mapped onto the ZX, YZ, and XY planes, respectively. However, the slices yielded limited information along the optical (Z) and high computing burden. Therefore, only the projection onto the XY plane was considered.

### Image preprocessing

According to the imaging technique (Van der Walt et al., 2014), the photomultiplier tubes (Ge et al., 2016) mainly introduced salt and pepper noise. Therefore, the 2D median filter was used to reduce noise and a partial differential equation (PDE) was used to enhance the image. Furthermore, in order to reduce the disturbance of the backbone extraction we used the opening top-hat and majority operator to eliminate small jiggers.

The original gray image *I*(*x*, *y*) and the structure elements (SE) *S*(*i*, *j*) were then set. The opening top-hat operator (Zhang et al., 2014) is defined as following:

(1) \begin{eqnarray*}I\circ S=(I\mrm{\Theta }S)\oplus S\end{eqnarray*}

in which *I*∘*S* is defined as function *I* executed the opening operation via SE ⊕ and Θ is defined as the dilation and erosion respectively.

The majority operator is defined as:

(2) \begin{eqnarray*}P= \left\{ \begin{array}{@{}l@{}} \displaystyle 1,\text{more than}\hspace*{1em}n\hspace*{1em}\text{positive pixels in its 3 by 3 window}\\ \displaystyle 0,\text{otherwise} \end{array} \right. \end{eqnarray*}

indicating that a pixel is considered to be part of the major line if there are more than *n* positive pixels in its 3*3 window. Otherwise, the pixel’s noise information must be relegated to 0.

### Ridge based backbone extraction

Along the pipeline of spine analysis the backbone detection is a critical step, underlining the importance of finding an effective and robust backbone detection algorithm. In this section we tried to use curvilinear structure detectors to find the dendritic backbone. One of the most well-known curvilinear structure detection methods is to use the principal curves proposed by Kegl & Krzyzak (2002) and Kegl et al. (2000), in which the curve is defined as a line passing through the middle of the data in a certain sense. However, this method was restrictive in that it assumed there would be single lines without self-intersection. Considering that the dendrite has multiple branches, we followed the detector that extracts the curvilinear structure from the data of ridge curves by the density estimation—so-called the ridge-based curvilinear structure detector. The multiple dendritic branches were detected on the assumption that the branches were separated by low density areas. The critical step then becomes the density estimation from the original image data. Here, we employed the nonparametric estimation directly from the samples represented by the observed variable *X* within a specific compact domain Ω*ϵR*_*d*_ . First, the image was normalized into binary image. We set *T* = 1 for the foreground clutter and *T* = 0 for the background clutter.

The probabilities

(3) \begin{eqnarray*}D(T=1)=\eta ,\hspace*{10.00002pt}\text{and}\hspace*{10.00002pt}D(T=0)=1-\eta \end{eqnarray*}

in which, *η* ∈ [0, 1]. We defined the generating function ${\{{f}_{i}\}}_{i=1}^{n}$, $i= \left\{ 1,2,3,3\ldots n \right\} $ with unit length of parameters, where *n* represents the number of the dendrite. ${\{{f}_{i}\}}_{i=1}^{n}$ depends on two parameters *i* ∈ {1, 2, 3, ...., *n*}, *θ* ∈ *D*_*i*_ where *D*_*i*_ is represented by the data samples along each dendrite. The output of *X* depends on function *f*_*i*_ and noise ε : *N*_*d*_(0, *σ*^2^). Density

(4) \begin{eqnarray*}P(I=i)={w}_{i}\end{eqnarray*}

in which *w*_*i*_ > 0, ∑*w*_*i*_ = 1.

(5) \begin{eqnarray*}{D}_{x}(x{|}T=1,I=i,\mrm{\Theta }=\theta )= \frac{1}{(\sqrt{2\pi }\sigma )^{d}} \exp \nolimits \left( - \frac{{ \left\| x-{f}_{i}(\theta ) \right\| }^{2}}{2{\sigma }^{2}} \right) .\end{eqnarray*}

We employ marginalized and the joint density to determine total density, which depends solely on the observed sample point *X*.

(6) \begin{eqnarray*}{D}_{x,T,I,}(x,T,i,\theta )& =& {D}_{x}(x{|}T=1,I=i,\mrm{\Theta }=\theta ){D}_{T,I,\mrm{\Theta }}(1,i,\theta )\nonumber\\\displaystyle & =& {D}_{x}(x{|}T=1,I=i,\mrm{\Theta }=\theta ){D}_{\mrm{\Theta }}(\theta {|}I=i){D}_{T,I}(1,i)\nonumber\\\displaystyle & =& {D}_{x}(x{|}T=1,I=i,\mrm{\Theta }=\theta ){D}_{\mrm{\Theta }}(\theta {|}I=i)D(I=i)D(T=1)\end{eqnarray*}

and

(7) \begin{eqnarray*}{D}_{x,T,I,}(x,0,i,\theta )={D}_{x}(x{|}T=0)D(T=0)\end{eqnarray*}

in which, *D*_*x*,*T*,*I*,_(*x*, 0, *i*, *θ*), and Θ represent the joint density and axis along the specified dendrite, respectively. When we summed the joint density over the domains of the discrete random variable *I* and *T* (subsequently integrated over the domain of the continuous variable Θ), marginal density (Alghalith, 2016) that provides the probabilities of various values of the variables in the subset without reference to the values of the other variables could be determined as:

(8) \begin{eqnarray*}{d}_{x}(x)= \frac{\rho }{(\sqrt{2\pi }\sigma )^{d}} \sum _{i=1}^{n}{w}_{i}\int \nolimits _{{D}_{i}}\exp \nolimits \left( - \frac{{ \left\| x-{f}_{i}(\theta ) \right\| }^{2}}{2{\sigma }^{2}} \right) {D}_{\mrm{\Theta }}(\theta {|}I=i)d\theta + \frac{1-\eta }{V(\mrm{\Omega })} \end{eqnarray*}

in which *V*(Ω) is the volume of domain Ω.

The kernel density estimation was then used, which could be determined from a set of samples $Y={\{{y}_{i}\}}_{i=1}^{N}\subset {R}^{2}$ of the probability density *D*:

(9) \begin{eqnarray*}\hat {D}= \frac{1}{N} \sum _{i=1}^{N}{K}_{H}(x-{y}_{i})\end{eqnarray*}

in which *K*_*H*_ is the Gaussian function (Cecchin et al., 2015) with a symmetric and positive kernel bandwidth in the domain *R*^2^ → [0, ∞], and *H* ∈ *R*^2×2^.

We assumed that the ridge was present in the area of high probability density. Thus, the dendrites were thought to be separated by a low density area. The ridge points (Micheal, Vani & Sanjeevi, 2014) were determined based on the principal that modes lie on a ridge curve. Accordingly, we first found modes of $\hat {D}$, and constructed the ridge point sets passing through these modes by using them as initial points. The algorithm is listed in the following steps:

**Step 1:** The modes of $\hat {D}$ are obtained by establishing the local maximum from each sample point.

**Step 2:** Step1 is iteratively applied to find the ridge curve components.

**Step 3:** The ridge curve segments are traced based on the following Eq. (10) adapted to the initial value as

(10) \begin{eqnarray*} \frac{\mathrm{d}}{\mathrm{d}\theta } \left( P(x(\theta )){\nabla }^{2}D(x(\theta )) \frac{\nabla D(x(\theta ))}{\parallel \nabla D(x(\theta ))\parallel } \right) =0,\hspace*{10.00002pt}x(0)={x}_{0}.\end{eqnarray*}

**Step 4:** A given point is projected onto the *n*-dimensional ridge set of $\hat {D}$, which is the density estimate.

**Step 5:** The ridge curve sets are separated by a low density area with a threshold of the density value *ϑ*.

The spurs caused by the connected spines were excluded based on the observation that there was only one exact main ridge curve structure passing through an intersection point. The proposed method spitted any other ridge curve components passing through such a point.

### Boundary location based on the density invariance

The local line direction was determined via the 2nd directional derivatives of the image (Zamani et al., 2012). Along the two directions perpendicular to the local line, the sharp difference of the density is thought to be the boundary of the dendrite based on the gray scale image.

The density of each pixel is given *I*(*p*) of point (*x*, *y*) in the original image, and *α* is the predefined pixel intensity value:

(11) \begin{eqnarray*}\text{if} \left\{ \begin{array}{@{}l@{}} \displaystyle I(p)\geq \alpha ,p\hspace*{1em}\text{belongs to the line pixel}\\ \displaystyle I(p)\lt \alpha ,p\hspace*{1em}\text{does not belong to the line pixel}. \end{array} \right. \end{eqnarray*}

### Hu moment invariants based feature extraction

In order to establish biological significance, it is essential to characterize the shape of the spinal structures. Spines are historically classified into three categories based on the shape of specific structures, including the spinal neck and head. Therefore, shape descriptors are critical for spinal classification and quantitative analysis. In this paper we employ a shape descriptor designated as the image moment. For a 2D Neuron image *I*(*x*, *y*), we define the raw moment of order (*p* + *q*) as

(12) \begin{eqnarray*}{M}_{pq}=\sum _{x}\sum _{y}{x}^{p}{y}^{q}I(x,y)\end{eqnarray*}

where *p*, *q* = 0, 1, 2, ….

**Central moment:** In practice, the central moments *μ* are usually utilized to replace the raw moment in Eq. (13)

(13) \begin{eqnarray*}{\mu }_{pq}=\sum _{x}\sum _{y}(x-\bar {x})^{p}(y-\bar {y})^{q}I(x,y)\end{eqnarray*}

(14) \begin{eqnarray*}\bar {x}= \frac{{M}_{10}}{{M}_{00}} ,\hspace*{20.00003pt}\bar {y}= \frac{{M}_{01}}{{M}_{00}} .\end{eqnarray*}

Central moments are translational-invariant.

**Normalized central moment:** When dividing the corresponding central moment by the properly scaled (00)th moment, Central moments can be extended to be both scale and translation invariant, The division was called normalized central moment.

(15) \begin{eqnarray*}{\eta }_{pq}= \frac{{\mu }_{pq}}{{\mu }_{00}^{ \left( \frac{p+q}{2} +1 \right) }} .\end{eqnarray*}

**Hu moment invariants:** In order to allow for invariable rotation the above moments were reformulated. There are two different methods for producing rotation moment invariants described by Hu & Mingkuei (1962). The first method is principal axes. However, when images do not have unique axes that are rotationally symmetrical, the first can be broken down. The second method is Hu moment invariants (HuMI). Hu derived these equations from algebraic invariants and applied them to the moment generating function under the condition of a rotation transformation. They consist of a set of nonlinear centralized moment (NCM) equations as

(16) \begin{eqnarray*}\begin{array}{@{}lll@{}} \displaystyle {H}_{1}&\displaystyle =&\displaystyle {\eta }_{20}+{\eta }_{02}\\ \displaystyle {H}_{2}&\displaystyle =&\displaystyle ({\eta }_{20}-{\eta }_{02})^{2}+4{\eta }_{11}^{2}\\ \displaystyle {H}_{3}&\displaystyle =&\displaystyle ({\eta }_{30}-3{\eta }_{12})^{2}+(3{\eta }_{21}-{\eta }_{03})^{2}\\ \displaystyle {H}_{4}&\displaystyle =&\displaystyle ({\eta }_{30}+{\eta }_{12})^{2}+({\eta }_{21}+{\eta }_{03})^{2}\\ \displaystyle {H}_{5}&\displaystyle =&\displaystyle ({\eta }_{30}-3{\eta }_{12})({\eta }_{30}+{\eta }_{12})[({\eta }_{30}+{\eta }_{12})^{2}-3({\eta }_{21}+{\eta }_{03})^{2}]\\ \displaystyle &\displaystyle &\displaystyle +~(3{\eta }_{21}-{\eta }_{03})({\eta }_{21}+{\eta }_{03})[3({\eta }_{30}+{\eta }_{12})^{2}-({\eta }_{21}+{\eta }_{03})^{2}]\\ \displaystyle {H}_{6}&\displaystyle =&\displaystyle ({\eta }_{20}-{\eta }_{02})[({\eta }_{30}+{\eta }_{12})^{2}-({\eta }_{21}+{\eta }_{03})^{2}]+4{\eta }_{11}({\eta }_{30}+{\eta }_{12})({\eta }_{21}+{\eta }_{03})\\ \displaystyle {H}_{7}&\displaystyle =&\displaystyle (3{\eta }_{21}-{\eta }_{03})({\eta }_{30}+{\eta }_{12})[({\eta }_{30}+{\eta }_{12})^{2}-3({\eta }_{21}+{\eta }_{03})^{2}]\\ \displaystyle &\displaystyle &\displaystyle -~({\eta }_{30}-3{\eta }_{12})({\eta }_{21}+{\eta }_{03})[3({\eta }_{30}+{\eta }_{12})^{2}-({\eta }_{21}+{\eta }_{03})^{2}]. \end{array}\end{eqnarray*}

It has been clearly observed that HuMI consist of absolute orthogonal (i.e., rotation) moment invariants which can be used for rotation, translation and scale invariant pattern recognition (Noronha & Nayak, 2013; Xiang et al., 2014; Žunic & Žunic, 2013).

### Twin SVM based spine classification

We did not use artificial neural network (Hernandez-Serna & Jimenez-Segura, 2014) since it may suffer from overfitting. The principle of the standard SVM involves setting up two parallel planes so that each is nearest to one of two datasets with the two planes being as far apart as possible (Kim et al., 2015; Modinos et al., 2013; Wu & Zhang, 2012; Zhang & Wang, 2015). The planes are described as

(17) \begin{eqnarray*}{w}^{T}x+b=0\end{eqnarray*}

which lie midway between the bounding planes provided by

(18) \begin{eqnarray*}{w}^{T}x+b=1\hspace*{10.00002pt}\text{and}\hspace*{10.00002pt}{w}^{T}x+b=-1.\end{eqnarray*}

The goal of the standard SVM is to maximize the margin in the form as

(19) \begin{eqnarray*}\begin{array}{@{}l@{}} \displaystyle {Min}_{w,b}\hspace*{10.00002pt} \frac{1}{2} {w}^{T}w\\ \displaystyle \text{subject to}\hspace*{1em}{A}_{i}w\geq 1-b\hspace*{1em}\text{for}\hspace*{1em}{y}_{i}=1\hspace*{1em}\text{and}\hspace*{1em}{A}_{i}w\geq 1-b\hspace*{1em}\text{for}\hspace*{1em}{y}_{i}=-1. \end{array}\end{eqnarray*}

Jayadeva, Khemchandani & Chandra (2007) proposed a Twin SVM (TSVM) that generated a pair of nonparallel planes for data classification, instead of two parallel planes via the standard SVM. However, in TSVM it was necessary to solve two quadratic programming problems, with one in SVM. Reports indicated that TSVM had better performance with regards to for classification and run time than did standard SVM.

The TSVM classifier is implemented by solving the following two quadratic programming problems

(20) \begin{eqnarray*}\begin{array}{@{}l@{}} \displaystyle {Min}_{{w}^{(1)},{b}^{(1)},q}\hspace*{10.00002pt} \frac{1}{2} (A{w}^{(1)}+{e}_{{}^{1}}{b}^{(1)})^{T}(A{w}^{(1)}+{e}_{1}{b}^{(1)})+{c}_{1}{e}_{2}^{T}q\\ \displaystyle \text{subject to}-(B{w}^{(1)}+{e}_{2}{b}^{(1)})+q\geq {e}_{2},q\geq 0 \end{array}\end{eqnarray*}

(21) \begin{eqnarray*}\begin{array}{@{}l@{}} \displaystyle {Min}_{{w}^{(2)},{b}^{(2)}q}\hspace*{10.00002pt} \frac{1}{2} (B{w}^{(2)}+{e}_{2}{b}^{(2)})^{T}(B{w}^{(2)}+{e}_{2}{b}^{(2)})+{c}_{2}{e}_{1}^{T}q\\ \displaystyle \text{subject to}-(A{w}^{(2)}+{e}_{1}{b}^{(2)})+q\geq {e}_{1},q\geq 0 \end{array}\end{eqnarray*}

in which *c*_1_, *c*_2_ > 0 are parameters, and *e*_1_, *e*_2_ are vectors of those with appropriate dimensions.

The TSVM sets up a hyper-planes for each class and classifies a sample according to which hyper-plane any given point is closest to relative to Euclidean distance. TSVM attempts to find the minimal squared distance based on Eq. (21) , which requires keeping the hyper-plane near to the points of one class. Simultaneously, a hyper-plane is required to be at a certain distance from the other class. Should the hyper-plane fall within predefined distances, a set of error variables is used to measure the error in the form of Eq. (19) . In order to improve the classification accuracy we employed the kernel nonlinear classifier Eqs. (22)–(23) to generate surfaces instead of planes as Eqs. (20)–(21).

(22) \begin{eqnarray*}\begin{array}{@{}l@{}} \displaystyle {{Min}}_{{U}^{(2)},{b}^{(2)},q}\hspace*{10.00002pt} \frac{1}{2} \left\| (K(B,{C}^{T}){u}^{(2)}+{e}_{2}{b}^{(2)}) \right\| ^{2}+{c}_{1}{e}_{1}^{T}q\\ \displaystyle \text{subject to}\hspace*{1em}(K(A,{C}^{T}){u}^{(2)}+{e}_{1}{b}^{(2)})+q\geq {e}_{1},q\geq 0 \end{array}\end{eqnarray*}

(23) \begin{eqnarray*}\begin{array}{@{}l@{}} \displaystyle {\mathop{\rm Max}}_{\gamma }{e}_{1}^{T}\gamma - \frac{1}{2} {\gamma }^{T}L({N}^{T}N)^{-1}{L}^{T}\gamma \\ \displaystyle \text{subject to}\hspace*{1em}0\leq \gamma \leq {c}_{2} \end{array}\end{eqnarray*}

in which $L= \left[ K(A,{C}^{T}),{e}_{1} \right] ,N= \left[ K(B,{C}^{T}),{e}_{2} \right] $.

The advantage of the TSVM is that it can be up to four times faster than the traditional SVM depending on computation size. In addition, TSVM has better generation than the traditional SVM.

## Experiment Results and Discussion

The experiments were performed on an IBM with a 3 GHz core i3 processor and 8 GB of RAM running a Windows 7 operating system. The algorithm was developed in-house using Matlab 2014a without optimization (MathWorks, Natick, MA, USA). The average runtime for a 1,024 × 1,024 image was 0.43s on a computer with a 3 GHz processor. This speed can be increased 10–100 times in C+ + with optimization.

### Backbone extraction and dendrite location

Figure 3 shows different cases of the backbone extraction based on the ridge detection and boundary location via the density difference. The purple color indicates the backbone, and the red color indicates the boundary. Figure 3A show the dendrite location result with multiple branches. Figures 3C and 3D show a single dendrite with one branch. Figures 3E and 3F show a single dendrite with another one branch dendrite. Figures 3G–3J respectively provide one single dendrite. Figure 3 proves that the backbone extraction based on the ridge detection and boundary location based on the density difference is effective.

**Figure 2:**
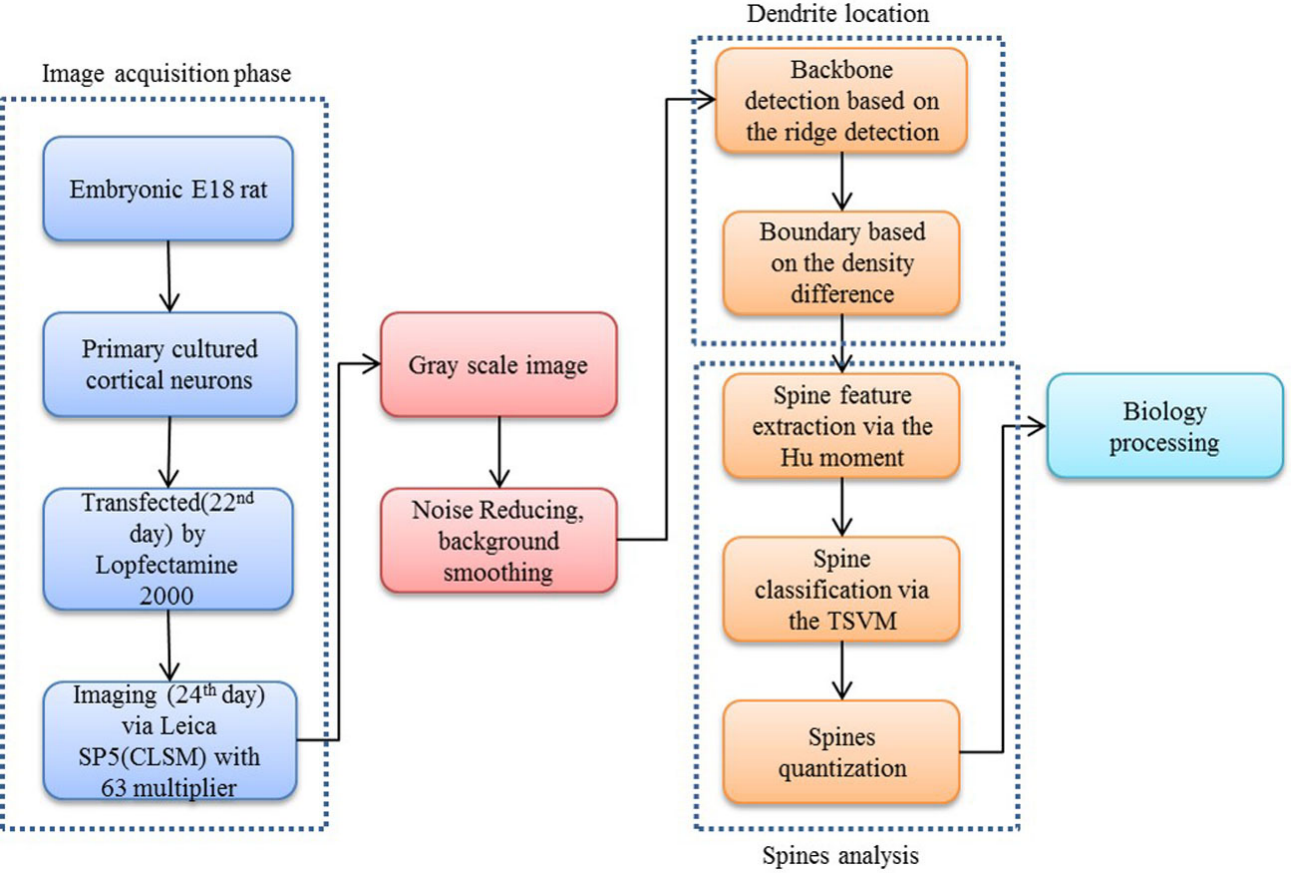
Flow chart of our proposed approach.

**Figure 3:**
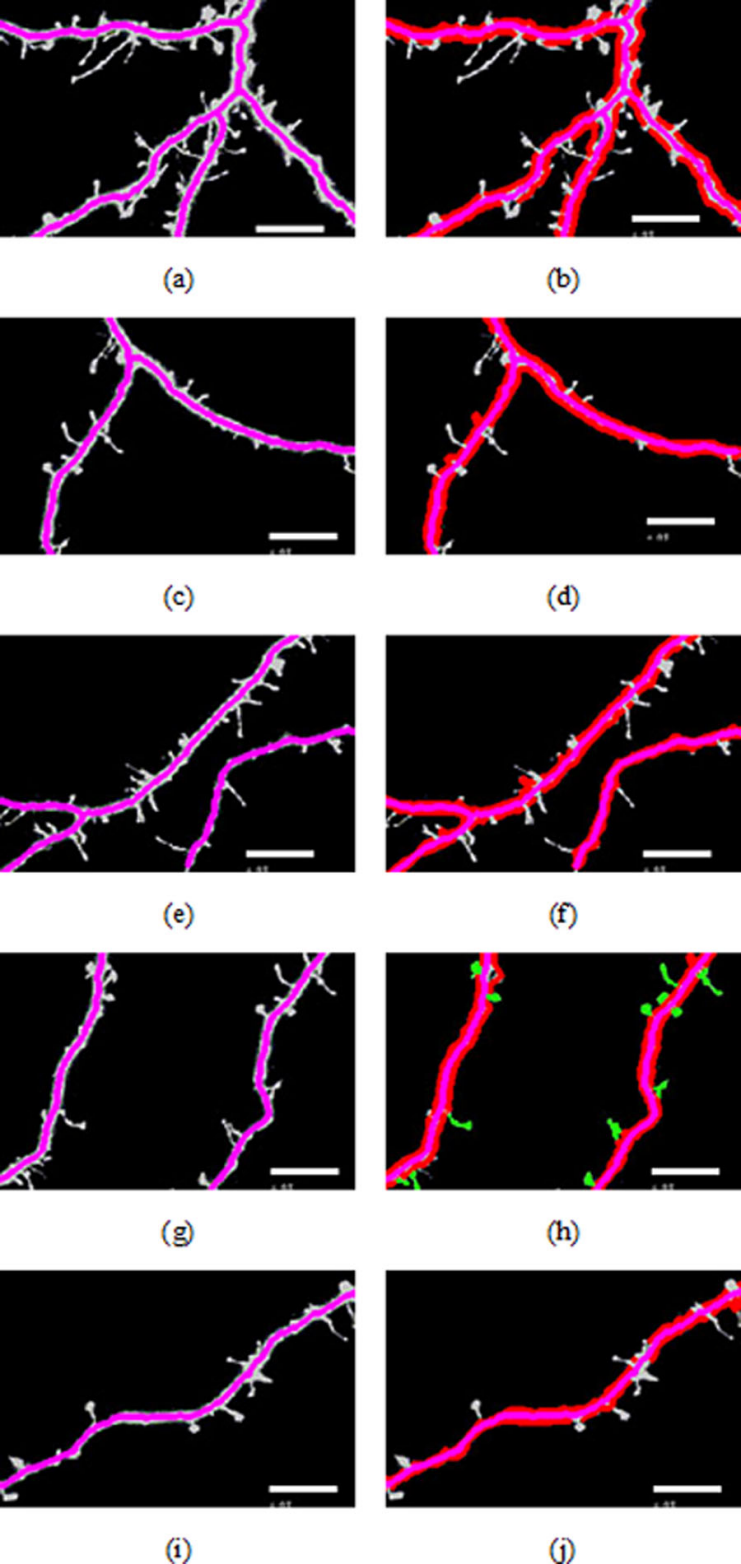
Results of backbone extraction and boundary location.

**Figure 4:**
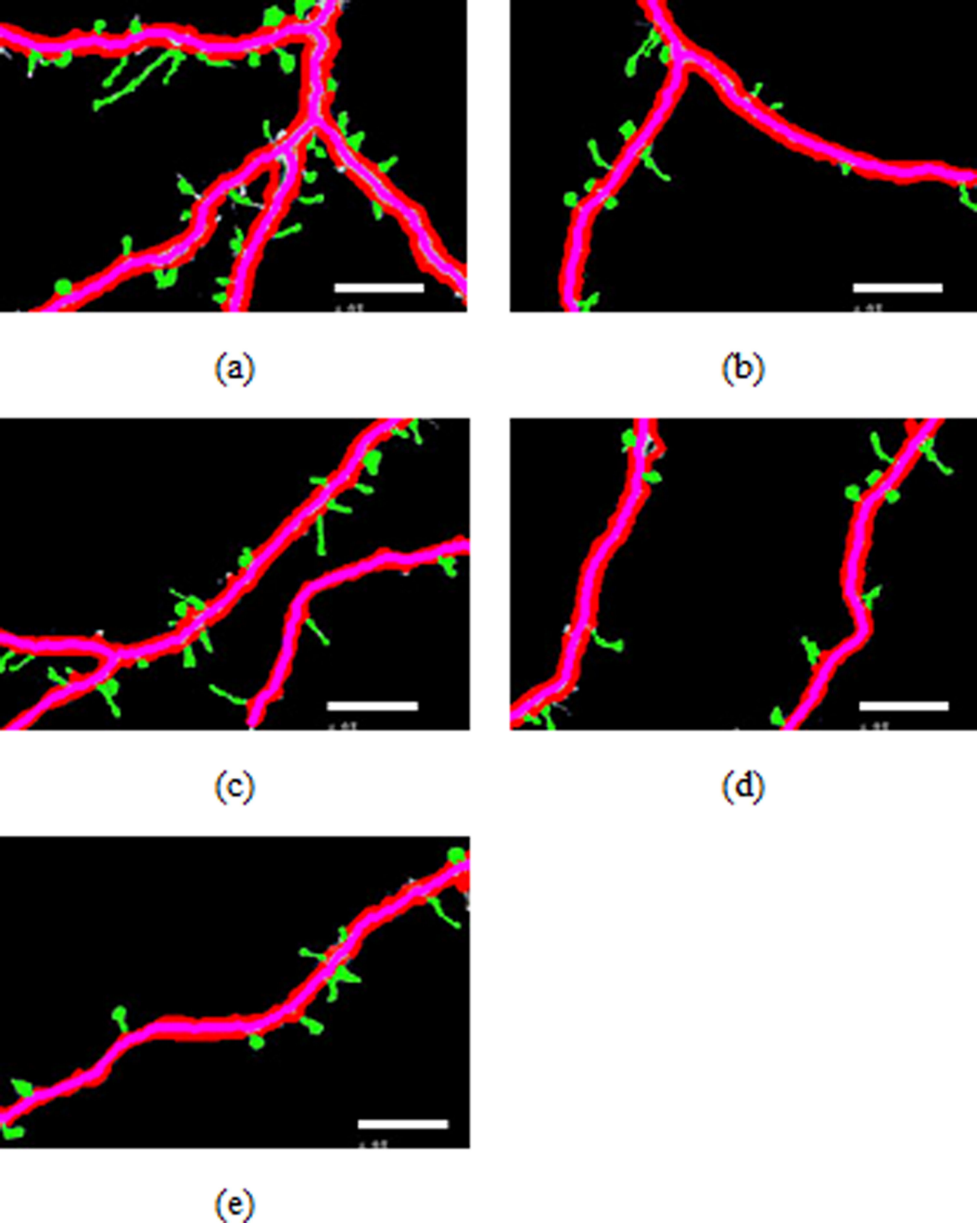
Spines detection results.

### Spine analysis

Figures 4A–4E illustrate the spine detection results of the sub-images for the condition in which that the dendrite backbone and boundary are marked. The pseudo spines were excluded based on the TSVM. After the dendrite and backbone were detected, our first step was to apply the Hu moment. We extracted the features of the remaining connected components and classified them as either true spines or pseudo-spines. Figure 4 shows two Examples of the pseudo spines, marked by a purple circle.

Table 1 shows the quantities data corresponding to Figs. 4A–4E. As we can find from Table 1, the total length of the dendrite, the number, total length and the area of the spines are recorded for the statistics analysis to reach the biology meaning.

**Table 1:** Quantities analysis of the dendrite and spiness.

	Total length of the dendrite (µm)	The number of the spine	Total length of the spines (µm)	Total area of the spines
Figure 4A	282.1	31	116	31.8
Figure 4B	160.2	13	37.9	9.6
Figure 4C	220.6	22	56.0	15.1
Figure 4D	166.2	13	46.0	12.8
Figure 4E	130.2	11	41.7	13.0

Figure 6 demonstrates that the area and length have similar distributions. For the normalization, *L* represents for largest value for the smallest area and *R* for the true value of the spine It is provided as:

(24) \begin{eqnarray*}N= \frac{R}{L-S} .\end{eqnarray*}

**Figure 5:**
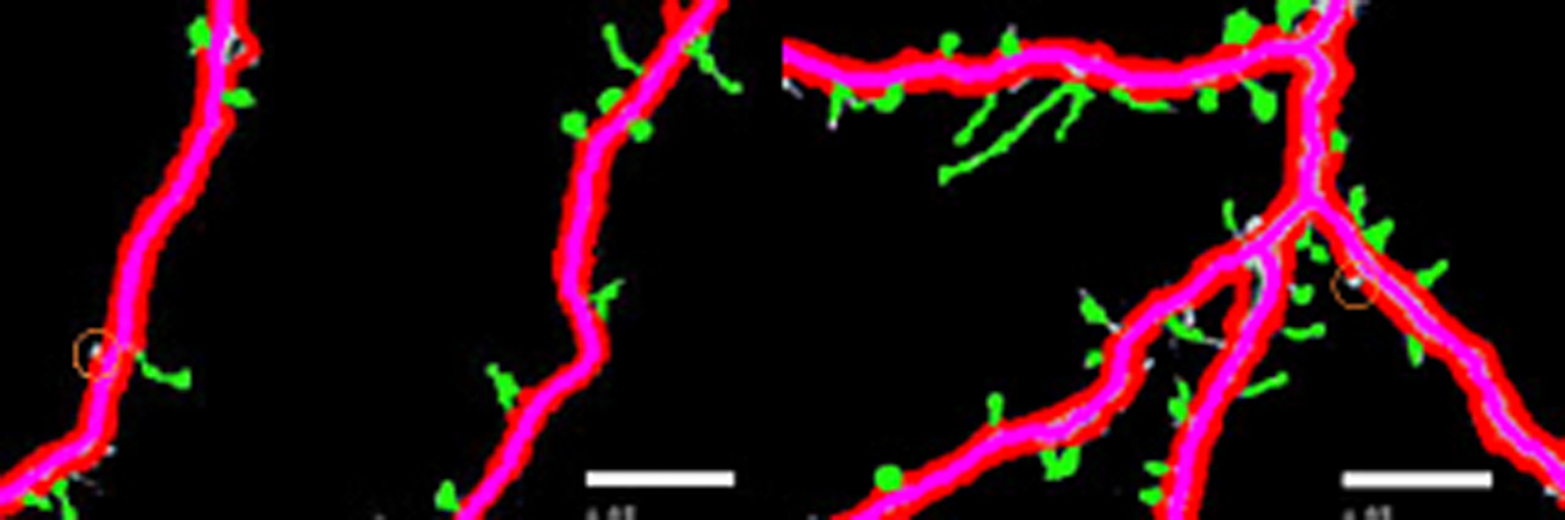
Examples of pseudo spines.

**Figure 6:**
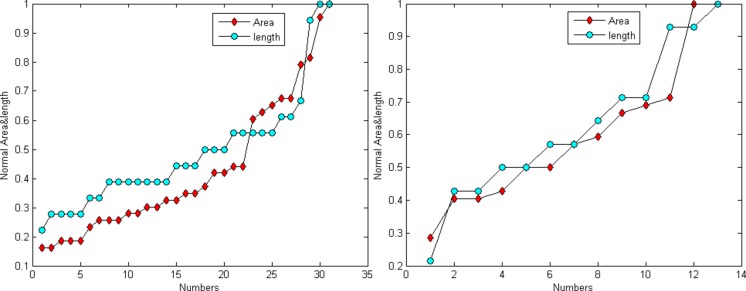
Distribution trend of the spine area and length of Figs. 4A and 4C.

Finally, The TSVM was utilized to classify the positive spines into predefined categories-MushRoom, Stubby and Thin-using the seven extracted Hu features. Table 2 shows the classification result for the pictures in Fig. 4. The average computation of the classification is 0.0012 s for 20 spines. The classification accuracy can reach 99%. Table 3 shows the Spine classification statistical results, we can find that the “MushRoom” type takes the vast majority of all the spines in our dataset followed by the “Thin:” types.

**Table 2:** Classification rate of spines.

	MushRoom	Stubby	Thin
Figure 4A	13	8	10
Figure 4B	4	4	5
Figure 4C	8	4	10
Figure 4D	6	3	4
Figure 4E	5	3	3

**Table 3:** Spine classification statistical results (%).

Spine type	MushRoom (%)	Stubby(%)	Thin(%)
RTSVM	46	22	32
Manual	39	24	37

**Figure 7:**
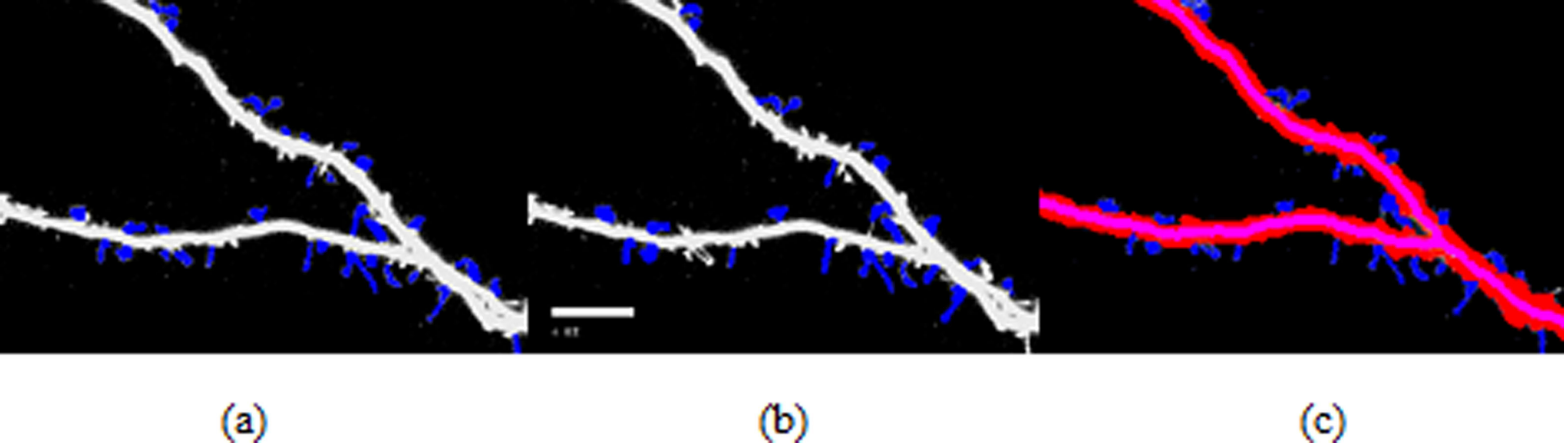
Spine detection result of ROI_ 1 via (A) Manual (B) Su’s method (C) RTSVM.

**Figure 8:**
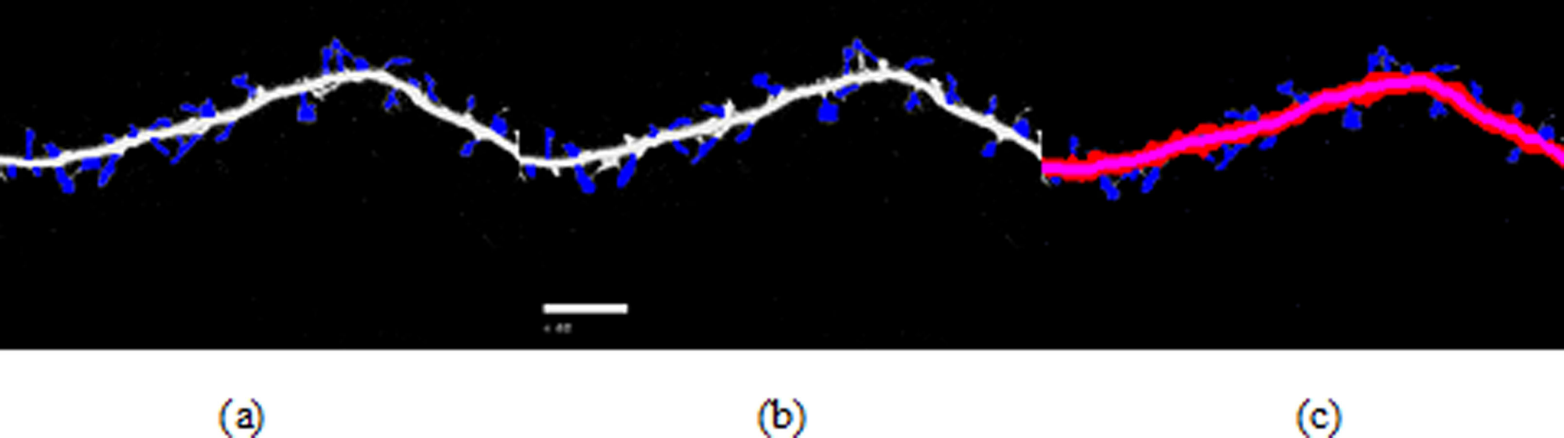
Spine detection result of ROI_ 2 via (A) Manual (B) Su’s method (C) RTSVM (our).

### Comparison of detection rate

We compared our proposed methods with Su’s algorithm and the manual mark. We randomly selected six images which include about 2,000 spines from the dataset and tested the accuracy. Figures 7 and 8 show two image examples to demonstrate the result of spine detection based on different methods, including via manual mark, Su’s method and our proposed RTSVM.

Table 4 shows the number of detected spines based on above three methods. For the first image (ROI_1), the detected number is 33, 25, and 29; for the second (ROI_2) is 28, 22, 23, respectively. It should be noted, however, that the manual method is vulnerable to mistaking as spines pixels belonging to the dendrite. Thus, it will have a much higher false alarm rate than the other methods. Conversely, Su’s method missed some positive spines due to the blurred dendrite boundary.

**Table 4:** Number of detected spine via Manual, Su’s method and RTSVM.

	Manual	Su’s method	RTSVM
ROI_ 1	33	25	29
ROI_ 2	28	22	23

### Comparison of the classification

We employed traditional support vector machine (SVM), generalized eigenvector proximal support vector machine (GESVM) and Twin support vector machine (TSVM) for the spine classification. Table 5 shows the classification result based on different SVMs for ROI_ 1 and ROI_ 2. We can find from Table 5 that GEPSVM and traditional SVM obtain similar classification results and are different from the results of TSVM. TSVM has better performance in the term of classification accuracy when compared to the ground truth (MushRoom 21, Stubby 12, Thin 19), which is provided by five experts in the neuroscience field who manually classified the spines into three predefined categories including “Mushroom,” “Stubby” and “Thin” according to the human vision by using Photoshop. For the conflict of the manual marking, the minority was supposed to subordinate to the major. Meanwhile, the computation time is much faster via TSVM than other two methods, which is 0.0015 s for the 52 spines instead of 0.007 s and 0.0054 s respectively by SVM and GESVM.

**Table 5:** Comparison of classification via different SVMs for ROI_ 1 and ROI_ 2.

	MushRoom	Stubby	Thin
SVM	18	10	24
GEPSVM	18	11	23
TSVM	20	13	19

Meanwhile, we compared our classification results based on the TSVM and Su’s algorithm. The result is shown in Table 6. We can find that our method obtain better classification result than Su’s algorithm (Su et al., 2014) based on the ground truth.

**Table 6:** Comparison of classification via TSVM and Sus algorithm for ROI_1_ and ROI_2_.

	MushRoom	Stubby	Thin
TSVM	20	13	19
Su’s algorithm	18	19	15

## Conclusion

This paper proposed a method based on the ridge detection for dendrite backbone and TSVM for the classification. The steps of the process were as follows: first a ridge curve was employed to extract the backbone of the dendrite. Then we used the sharp density difference to locate the boundary of the dendrite and the presumed starting points of the spines. Then TSVM classifier was built based on the seven spine features extracted by Hu moments invariant. The experiment demonstrated that the system is effective and efficient. The backbone extraction results show that the ridge detection performs well for curvilinear structures in the context of noisy data. We classified spines in terms of pseudo- vs. true spines, and also in terms of predefined categories of spines. In both cases, TSVM performed better than standard SVM and GEPSVM and demonstrated faster running times.

A major contribution of this paper is the development of a robust and efficient system for dendrite and spines analysis. Given that statistical analysis of neuronal structure is important for advancement in the fields of biology, and that large amounts of data are available due to advancements in imaging techniques, such a system is invaluable.

A limitation of this paper is that did not obtain data from all kinds microscopy images. In theory, the proposed system should work well for all types of microscopy. A second limitation is that the resolution is defective. Further improvements in imaging techniques will enhance the performance of our system.

We plan to continue our experiments, collect more data from different types of microscopy, and build a large database which can be used for the test of our system. We also need to optimize our algorithm to further improve accuracy and speed by latest swarm intelligence methods, such as biogeography-based optimization (Ji et al., 2015) and particle swarm optimization (Ji, Zhang & Wang, 2015). A human interface is also essential for the wide application of the system. New partial differential equations (Lopez Corona et al., 2014) are to be tested.

##  Supplemental Information

10.7717/peerj.2207/supp-1 Supplemental Information 1 Spine Detection

10.7717/peerj.2207/supp-2 Supplemental Information 2 Spine Detection 2

## Additional Information and Declarations

### Data Availability

The following information was supplied regarding data availability: The raw data has been supplied as Supplemental Dataset.
