# Supplementary figures and images for: Morphological analysis of dendrites and spines by hybridization of ridge detection with twin support vector machine
Source: PeerJ. 2016 Jul 20;4:e2207. doi: 10.7717/peerj.2207 (PMC4958009; doi:10.7717/peerj.2207)

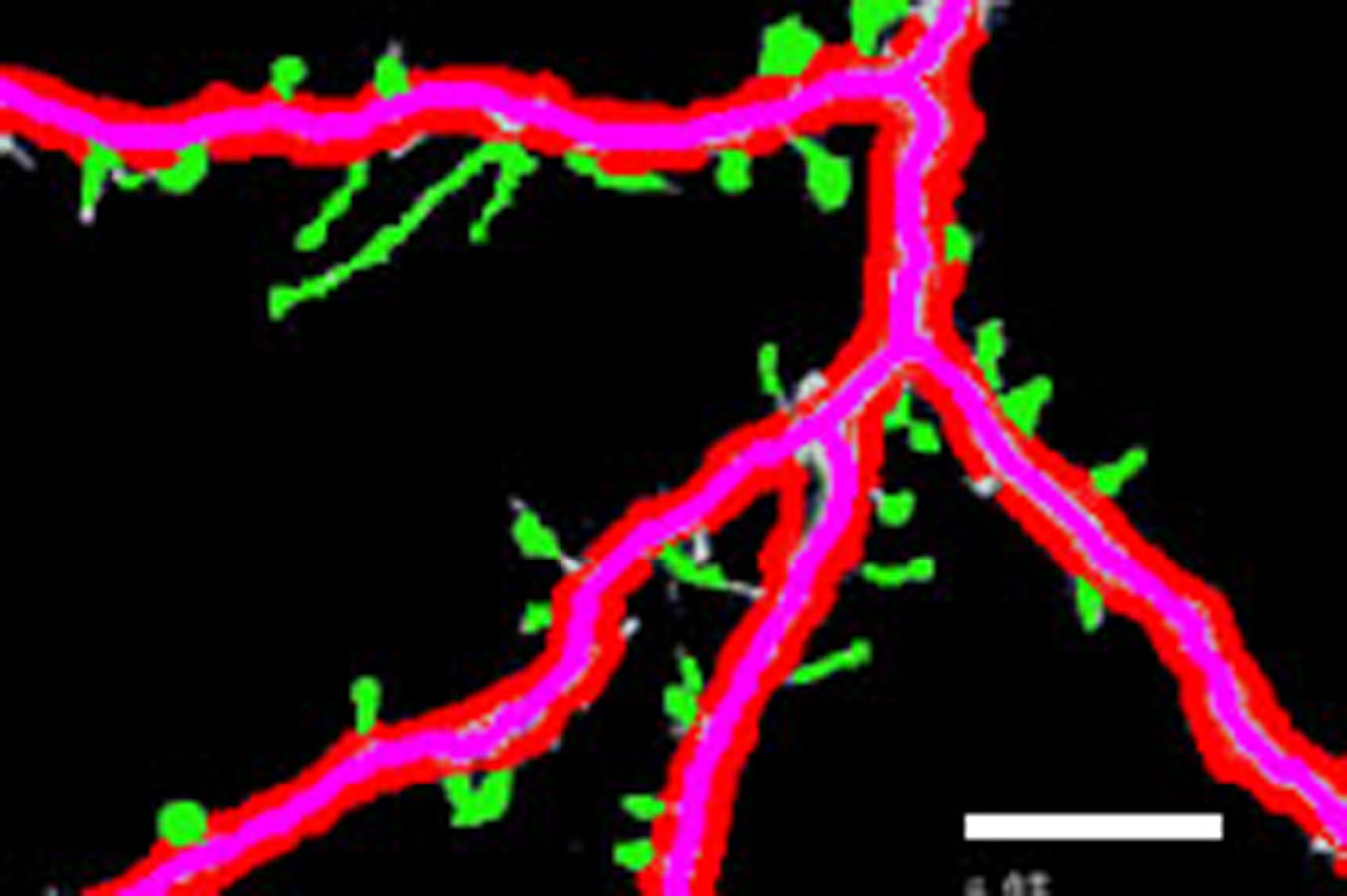

Supplement: Supplemental Information 1 [file peerj-04-2207-s001.jpg]

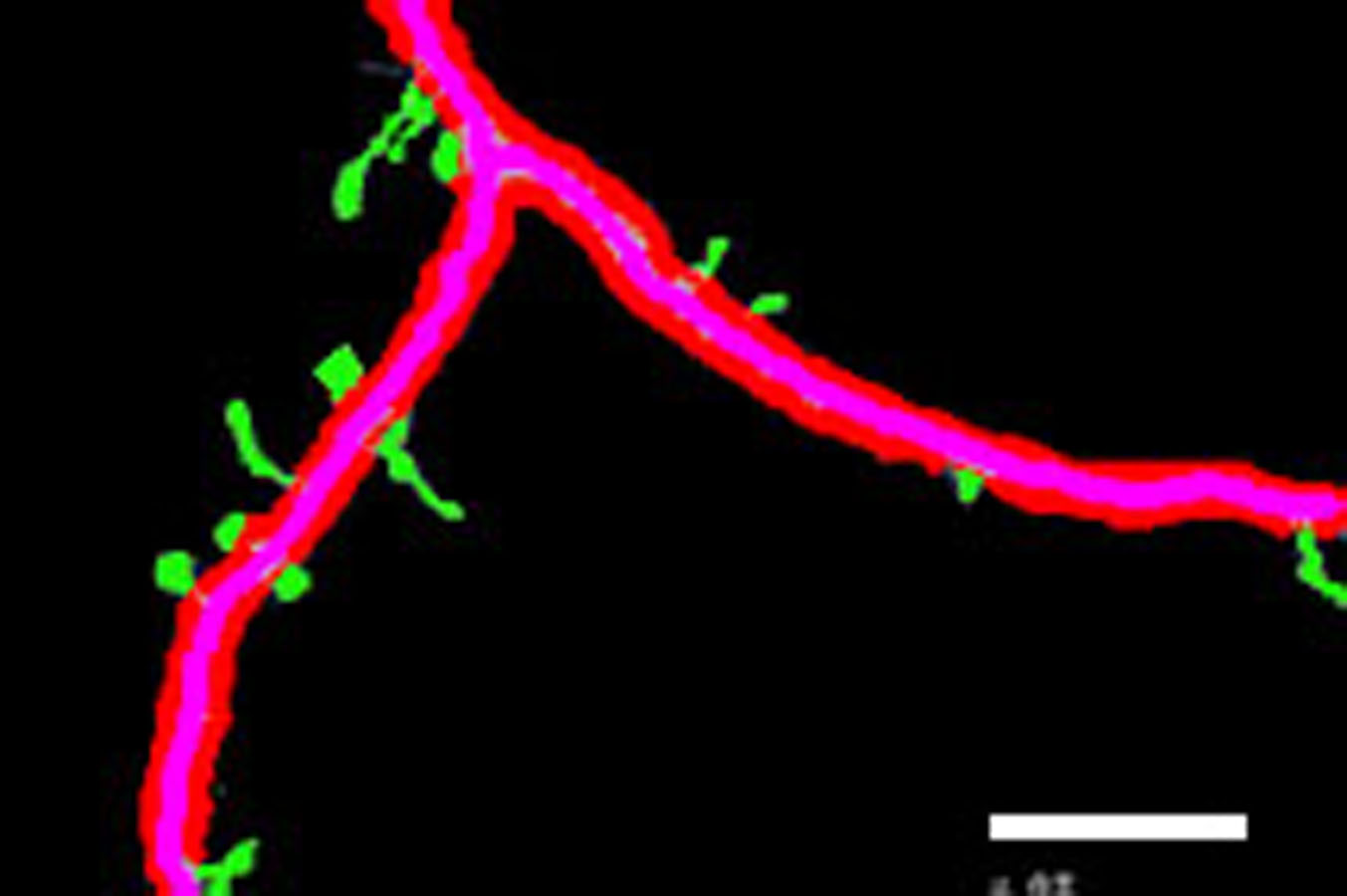

Supplement: Supplemental Information 2 [file peerj-04-2207-s002.jpg]
